# Biotic interactions in soil and dung shape parasite transmission in temperate ruminant systems: An integrative framework

**DOI:** 10.1002/eap.2956

**Published:** 2024-03-01

**Authors:** Christopher J. Boughton, Lesley T. Lancaster, Eric R. Morgan

**Affiliations:** ^1^ School of Biological Sciences, Queen's University Belfast Belfast UK; ^2^ School of Biological Sciences University of Aberdeen Aberdeen UK

**Keywords:** agroecology, agroecosystems, biocontrol, parasite control, sustainable agriculture

## Abstract

Gastrointestinal helminth parasites undergo part of their life cycle outside their host, such that developmental stages interact with the soil and dung fauna. These interactions are capable of affecting parasite transmission on pastures yet are generally ignored in current models, empirical studies and practical management. Dominant methods of parasite control, which rely on anthelmintic medications for livestock, are becoming increasingly ineffective due to the emergence of drug‐resistant parasite populations. Furthermore, consumer and regulatory pressure on decreased chemical use in agriculture and the consequential disruption of biological processes in the dung through nontarget effects exacerbates issues with anthelmintic reliance. This presents a need for the application and enhancement of nature‐based solutions and biocontrol methods. However, successfully harnessing these options relies on advanced understanding of the ecological system and interacting effects among biotic factors and with immature parasite stages. Here, we develop a framework linking three key groups of dung and soil fauna—fungi, earthworms, and dung beetles—with each other and developmental stages of helminths parasitic in farmed cattle, sheep, and goats in temperate grazing systems. We populate this framework from existing published studies and highlight the interplay between faunal groups and documented ecological outcomes. Of 1756 papers addressing abiotic drivers of populations of these organisms and helminth parasites, only 112 considered interactions between taxa and 36 presented data on interactions between more than two taxonomic groups. Results suggest that fungi reduce parasite abundance and earthworms may enhance fungal communities, while competition between dung taxa may reduce their individual effect on parasite transmission. Dung beetles were found to impact fungal populations and parasite transmission variably, possibly tied to the prevailing climate within a specific ecological context. By exploring combinations of biotic factors, we consider how interactions between species may be fundamental to the ecological consequences of biocontrol strategies and nontarget impacts of anthelmintics on dung and soil fauna and how pasture management alterations to promote invertebrates might help limit parasite transmission. With further development and parameterization the framework could be applied quantitatively to guide, prioritize, and interpret hypothesis‐driven experiments and integrate biotic factors into established models of parasite transmission dynamics.

## INTRODUCTION

Parasitic helminths have critically important negative impacts on animal productivity and health in grazing livestock systems. For the past 50 years or more, chemical control (anthelmintic medications) has been the principal means of regulating these parasites; however, inherent genetic diversity plus strong selection pressures are leading to evolved resistance to current chemical control measures, resulting in widespread ineffectual control (Rose Vineer et al., 2020). Simultaneously, other pressures are contributing to a change in priorities for agricultural management, including consumer demand for chemical‐free production lines and concerns around the environmental impact of chemicals and the maintenance of carbon storage (Blanckenhorn et al., 2013; Dignac et al., 2017; Goodenough et al., 2019; Verdú et al., 2015; Wall & Strong, 1987). This development demonstrates the need to consider and understand other options for limiting parasite populations. While a lot is known about the effects of abiotic factors, especially climate and weather (Charlier et al., 2020; van Dijk & Morgan, 2012), when it comes to helminth epidemiology, far less is understood about the biotic factors that impact parasite transmission. These factors are fundamental to parasite transmission dynamics within a wide array of contexts for both farmed and wild ruminants (Nichols et al., 2017), whereby modulation of the environment by other organisms can alter transmission risk, including of drug‐resistant strains (Brown et al., 2022). Importantly, though species may differ between agricultural and natural landscapes, the universal mechanisms of action undertaken by faunal communities remain similar (Hoeffner et al., 2021), indicating that understanding faunal interactions with parasites may have broad application. This may be particularly pertinent within the context of future sustainable agricultural solutions, as global change and altered farm management strategies shift the environmental context of the free‐living life cycle stages of parasites (Cable et al., 2017). A synthetic framework that encapsulates current knowledge, while highlighting unknowns, could direct the inclusion of community ecology in our understanding of livestock parasite life cycles, with the aim of considering and enhancing parasite management through manipulation of the natural environment. Gastrointestinal helminths are ripe for this approach, not only because their management is rapidly proving unsustainable due to drug resistance and environmental concerns, but also because their free‐living stages are closely associated with dung and soil and open to many biotic interactions that are known but poorly characterized and could provide a pathway to alternative control strategies.

Parasitic helminth life cycles comprise several free‐living stages, each of which provides opportunities for different biotic interactions to develop. For most gastrointestinal nematodes (GINs), for instance, especially the trichostrongylids that dominate in grazing ruminants and strongyles in equids, eggs are excreted in the feces where they hatch and begin their first two larval stages (L1 and L2) by feeding on bacteria within the feces and molting their cuticle between each stage (Zajac, 2006). These dung‐based larval stages could therefore be impacted by other organisms that feed on, live in, or utilize livestock dung for their own life cycles (Bacher et al., 2018; Holter, 1979; Pecenka & Lundgren, 2018). The third larval stage (L3) of GINs is the infective stage that migrates from the feces with a limited supply of energy to herbage, where it must be consumed by a suitable host in order for transmission to occur and the life cycle to continue. Organisms that are localized to or transiently visit the dung, soil, and/or herbaceous vegetation could therefore impact L3 parasite availability through consumption, hindering, or enhancing movement or exposing the larvae to more or less favorable conditions (Xie et al., 2020; Yang et al., 2020). If consumed by the host, GINs then undergo various within‐host stages before producing eggs to be excreted in the feces. A large proportion of the GIN life cycle occurs outside of the host, where it is exposed to other soil fauna within the grassland ecosystem, which may be capable of influencing the parasite population. Trichostrongylid lungworms with a direct life cycle such as *Dictyocaulus* spp. have a very similar life cycle to GIN in the free‐living stages. Eggs of other helminths, including liver and rumen fluke (e.g., *Fasciola* and *Calicophoron* spp.), tapeworms (e.g., *Moniezia* spp.), and some GINs (*Nematodirus* spp.), do not hatch in the dung but rely on dung degradation to reach the soil, where they develop further. This review considers only the dung and soil compartments and not the subsequent complex life cycle stages of flukes, tapeworms, and protstrongylid lungworms, which involve invertebrate intermediate hosts and, hence, a multitude of additional biotic interactions. Furthermore, grazing by ruminants or other organisms can adjust the microclimate to which developmental stages of helminths are exposed (Khadijah et al., 2013), but this is also not considered further.

As resistance to many anthelmintic drugs becomes more prevalent, farmers are increasingly seeking alternative control methods for parasite management, such as rotational grazing, alternative forages and pasture management practices like mixed grazing (Bambou et al., 2021; Grace et al., 2019; Kumar et al., 2013; Velde et al., 2018), and biological control (Szewc et al., 2021). Underpinning the effectiveness and usage of many of these alternative strategies, however, are the fundamental biotic and abiotic interactions that occur on pasture. Unexplored biotic interactions could lead to unexpected effects on parasite transmission, explain variation in the success of control strategies in different environments, and modify outcomes from biocontrol or bioaugmentation using species known to prey on helminths. Studies on the biological control of GINs using nematophagous fungi or other organisms have typically focused on single species or combinations of closely related species administered in controlled conditions (Szewc et al., 2021), without considering ecological interactions with species already present in the grazing system.

Abiotic factors, including temperature and moisture, are known to strongly affect helminth development, for example through GIN larval hatching times, migration, and survival (Pandey et al., 1989; Young et al., 1980). Temperature and rainfall are also known to have effects on earthworms, fungi, and dung beetles (Fernández et al., 1999; Singh et al., 2016; Vessby, 2001), which are themselves also linked to GIN transmission success (Fernández et al., 1999; Grønvold, 1987; Leathwick et al., 2011; Sands & Wall, 2017; Waghorn et al., 2002). Further to this, changes in rainfall patterns and increased mean global temperatures due to climate change may provide conditions that are favorable to one species over another (Bellard et al., 2012; Singh et al., 2019; Tocco et al., 2021). Consequently, the change in climate could exert direct impacts upon each species within an ecosystem in combination with indirect impacts through the alteration of competition, other interspecies dynamics, or resource availability (Singh et al., 2021; Thieltges et al., 2008; Tocco et al., 2021). However, biotic and abiotic factors are not traditionally considered together when designing biocontrol or pasture management strategies to limit GINs or other helminths, an omission that may limit their effectiveness. Understanding how the interlinking biotic and abiotic factors combine to affect parasite transmission is fundamental if sustainable management strategies are to be established in the future. Here we aim to produce a schematic framework based upon the current literature that considers the functional impacts of different soil fauna on parasite transmission within grassland systems, focusing on temperate grasslands grazed by domestic ruminants. This focus is intended to limit the work to a coherent and manageable subset of livestock and parasite species and dung‐breeding fauna, as well as interactions with climate, but it could be repeated for other biomes including tropical pastures. The schematic aims to exemplify how one might incorporate community ecology into parasite transmission, to consider the role that dung and grassland ecosystems may have in facilitating or constraining environmental parasite stages, and to underpin rigorous exploration of nature‐based solutions for sustainable parasite management.

## A CONCEPTUAL FRAMEWORK FOR ECOLOGICAL INTERACTIONS WITH ENVIRONMENTAL PARASITE STAGES

**FIGURE 1:**
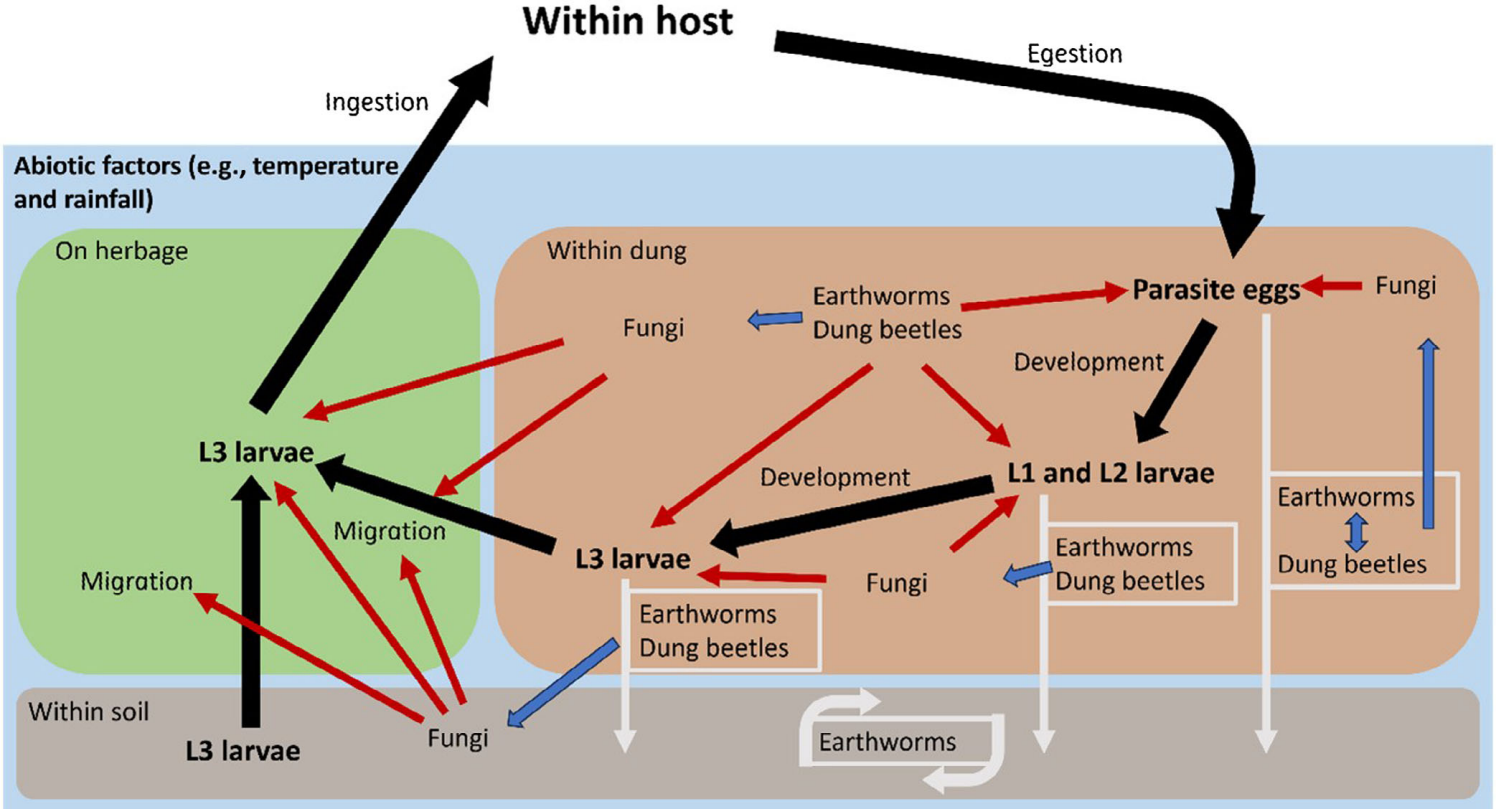
Schematic diagram depicting influence of key soil fauna on parasite transmission. Compartments are detailed with relevant life cycle stages of the parasite. Black arrows indicate the progression through the environmental stages of the gastrointestinal nematode life cycle. Three key soil fauna groups are considered in the schematic: earthworms, dung beetles, and fungi. White arrows indicate movements between environmental compartments with associated organisms detailed within white box. Curved white arrows describe movement within compartment, with corresponding organisms contained within. Red arrows indicate a negative impact on the vital rates of that stage of the parasite life cycle, with the soil fauna that causes this impact labeled. Some active unassisted movement of gastrointestinal nematode (GIN) L3 between the dung, soil subsurface, and herbage does occur (Rose et al., 2015), but this figure considers the deeper soil layers. L1 and L2 refer to GIN specifically; eggs include GIN and other helminths.

A conceptual framework that considers the environmental stages of the typical life cycle of GINs, as well as stages of other helminths in the dung and soil and their interaction with three major groups of soil fauna, is presented in Figure 1. These three groups are selected for consideration due to their ecological association with dung and soil as consumers, transporters, or inhabitants, as well as for their potential role in biocontrol and nature‐based solutions for parasite transmission. The framework considers faunal interactions at different stages of the parasite life cycle, each of which are localized to different compartments of the environment. Crucially, each component of the schematic should not be viewed in isolation but instead presents a dynamic ecosystem where one biotic factor can influence another within and across compartments.

Information on soil fauna interaction effects with parasite life cycle stages was collected using a systematic literature review (Box 1) and is summarized below. Interactions between each group and parasites are considered first, followed by interactions between these groups themselves and system interactions, then the effects of climate and weather on them. Throughout, the emphasis is on processes that could impact parasite transmission, including but not limited to predation within the dung, altering the biophysical environment of the dung in ways that affect parasite development and survival, moving dung into soil, and facilitating migration of infective parasite stages from dung onto herbage.

BOX 1 **Systematic review search criteria. Papers were retrieved from Web of Science. Search strings for each factor are shown under the appropriate factor heading. General terms remained consistent within every search conducted, with reviews excluded from searches. Specific terms were combined to search selectively. Papers were selected if they were a primary piece of literature, data on the interaction could be disentangled if part of a larger investigation, and the paper detailed or quantified the interaction.**

**General:**

**Life stage terms:**

(Migration OR survival OR development OR transmission OR decomposition)

**Community terms:**

(Ecolog* OR interact* OR free‐living stage* OR herb* OR soil* OR grass* OR enviro* OR dung OR fecal* OR faece* OR feces)

**Environment terms:**

(Livestock* OR Cattle* OR cow* OR Sheep OR ruminant* OR goat OR horse OR bovine OR equine OR ovine OR pasture OR feces* OR dung* OR faece* OR fecal*)

**Specific:**

**Parasite terms:**

(Ostertagia OR Cooperia OR Teladorsagia OR Trichostrongyl* OR Haemonchus OR Dictyocaulus OR trematod* OR Nematodirus OR Fasciola OR Moneizia OR parasit* OR gastrointestinal OR GIN OR fluke OR lungworm OR strongy*)

**Fungi terms:**

(Fung* OR Duddingtonia OR Beauvaria OR nematoph* OR Monacrospor* OR Harprospor* OR Pilobolus OR Arthrobot* OR Clonostach* OR Pochonia* OR Mucor*)

**Earthworm terms:**

(earthworm* OR earthworm cast OR Aporrectodea OR Allolobophora OR Lumbricus OR Eisenia)

**Dung beetle terms:**

(Dung beetle* OR dung‐beetle* OR dweller* OR tunneller* OR Geotrupid* OR Scarab* OR Melinopt* OR Onthophag* OR Aphodiin* OR Aphodius OR Acrossus OR Geotrupes* OR Cercyon* OR Megasternum* OR Sphaeridium* OR Histeridae OR Philonthus*)

**Driver terms:**

(Climate change OR Climate* OR global warming OR temperature* OR moisture OR humidi* OR pH OR abiotic)

### Literature evaluation to explore parasite interactions with different taxa

**FIGURE 2:**
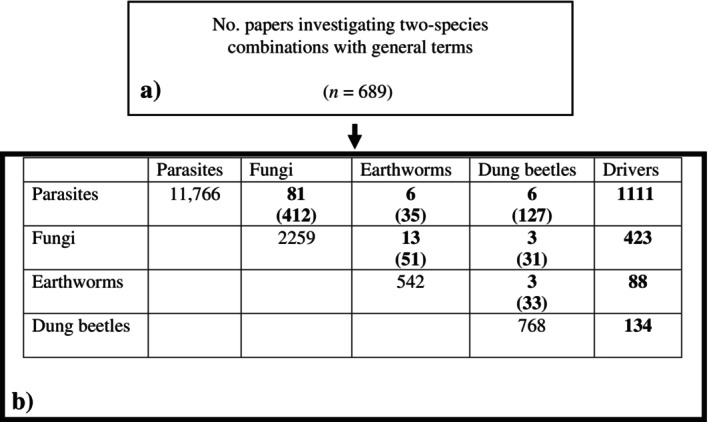
Schematic diagram displaying selection of eligible papers from systematic review. Number of total papers investigating the general terms and two‐species combinations (a). The table considers the number of papers investigating each combination, with the total number of accepted papers that explore the interaction on the top line, followed by the number initially returned in the bracket below (b). The driver column shows the number of papers initially returned for each soil fauna group with the abiotic driver terms included (Box 1).

**FIGURE 3:**
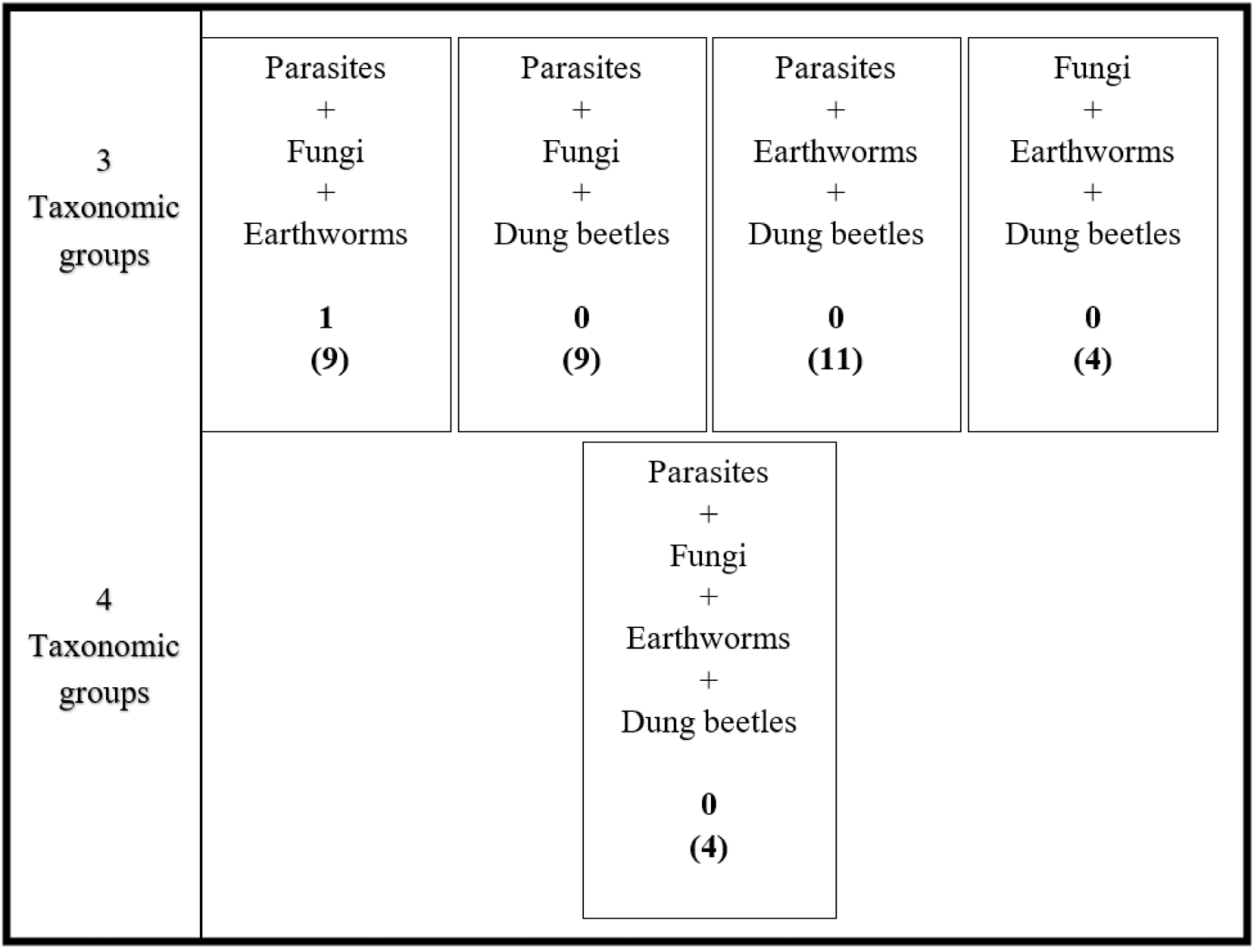
Schematic diagram detailing number of papers that investigate combinations of three or more taxonomic groups using general terms (Box 1). The diagram displays the total number of papers returned for each three or four species combination searches, alongside the general terms, in parentheses. The number of papers that investigate those interactions are above, that is, the figure not in parentheses.

**FIGURE 4:**
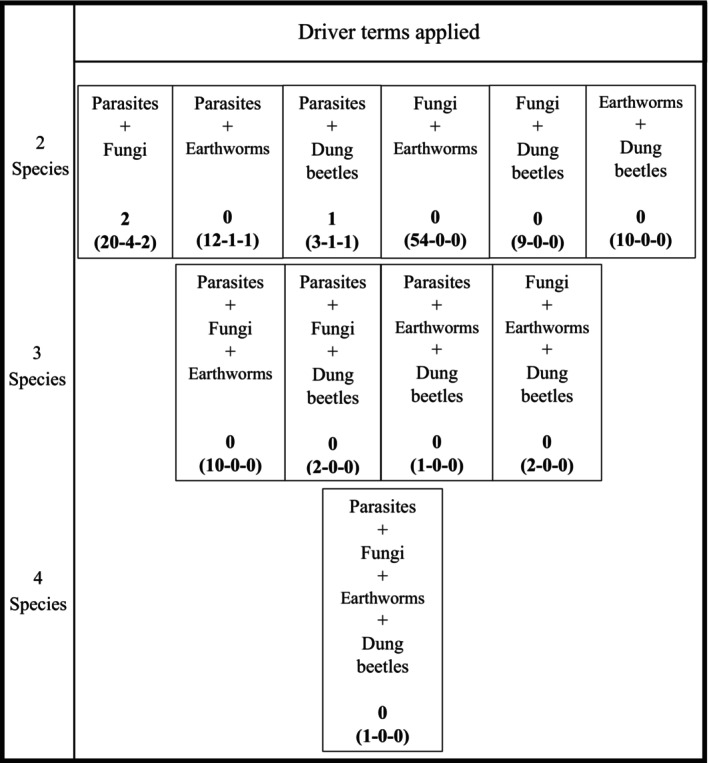
Schematic diagram detailing number of papers that investigate combinations of at least two species with general and driver terms. The diagram displays the number of papers returned for each two, three, or four species combination searches, alongside the general terms and driver terms. The number of papers returned in the search is given in parentheses, and the number that specifically investigate the interactions is above, that is, not in parentheses.

Literature searches were undertaken to evaluate current knowledge on different combinations of organisms within the temperate grassland system. Searches were constructed by aggregating search terms and subsequently screening returned papers to ensure that interactions could be detailed and quantified from the investigation, the paper was a primary source of literature, and the interaction could be interpreted if it was part of a larger interaction network (Box 1). Searches were nested and progressively more complex, starting at interactions between two taxonomic groups and increasing to four groups (Figures 2 and 3). Abiotic factors, including temperature and moisture, were then combined into the same search criteria to elucidate what is known about how the same interactions transpire under varying conditions and considering a changing climate (Figures 2 and 3). Papers from the searches were read at the title, abstract, and then full paper level, with progression to the subsequent levels accepted if they were a source of primary data and appropriately considered the relevant interactions. This search found that no returned papers effectively investigated the interaction between three or more soil fauna groups with respect to the abiotic factors (Figure 4). This suggests that ecological networks associated with GIN parasite life cycles, when considered as a whole system, are seldom explored despite individual interaction effects being known.

### Interactions between parasites and different taxa

#### Fungi and parasites

Fungi that feed on nematodes occur naturally in the environment and rapidly colonize dung pats (Hay et al., 1997; Kelly et al., 2009; Su et al., 2007). The most important process is increased mortality of preinfective and infective parasite stages, which is observed as a reduced proportion of eggs surviving and developing successfully to the infective stage. Of the taxonomic groups considered in this review, these nematophagous fungi show most potential for biocontrol due to their often strong negative impacts on parasitic nematode development success and the ability to augment fungal populations in dung by feeding fungal spores to animals. While this promise has been realized in the form of commercial feed supplements, the level of impact on parasites varies widely. Most trials have measured parasitic larval yield as a function of fungal augmentation without explicitly considering additional factors such as existing fungal communities and other features of the dung environment, which could be important to outcomes.

Fungus–parasite interactions occur readily within the natural environment, and trophic interactions have been categorized into four major groups based on the method of fungal predation on nematode communities: (1) ovicidal fungi, (2) endoparasitic fungi, (3) nematode‐trapping fungi, and (4) toxin‐producing fungi (Jiang et al., 2017; Nordbring‐Hertz et al., 2011; Zhang et al., 2020). Due to the abundance and diversity of nematophagous fungi within terrestrial ecosystems, many fungal species have been exploited as a method of biocontrol against agriculturally relevant parasite populations (Ahmed et al., 2014; Arroyo et al., 2016; Eysker et al., 2006; Paraud et al., 2005; Szewc et al., 2021; Zhang et al., 2020). This method of biocontrol relies on the augmentation and enhancement of naturally occurring processes within the grassland ecosystem, with the intention of supporting and benefitting livestock managers.

Ovicidal fungi are one of the fungal groups that have been explored less with respect to animal parasitic nematodes. Arroyo et al. (2017) examined the interaction between the ovicidal fungus *Mucor circinelloides* and the trematode rumen fluke *Calicophoron daubneyi*, applying fungal spores to the trematode within petri dishes, within the feces, and within aqueous test tube environments. They found a significant reduction in the percentage of successfully embryonated rumen fluke eggs compared to the control across all tested environments (Arroyo et al., 2017). Hernández et al. (2017) showed that ovicidal fungi can adhere to and penetrate the eggs and permanently damage the inner embryo. Some studies have considered the versatility of ovicidal fungi in damaging a wide variety of helminth species (Araújo & Salcedo, 1995; Braga et al., 2007), although those with other feeding styles have been developed further for application in biocontrol.

Endoparasitic fungi live by feeding on nematodes following adhesion to the nematode cuticle or ingestion of spores by the nematode (Balyoi et al., 2011). Most research into this trophic guild has used the fungal species *Clonostachys rosea*, due to its voracious and diverse consumption of nematode species (Rodríguez‐Martínez et al., 2018). Variable levels of reduction in nematode viability have been reported (Ahmed et al., 2014; Silva, Braga, Mendoza‐de‐Gives, Millán‐Orozco, et al., 2015; Silva et al., 2017), both through the addition of fungi to dung containing parasitic nematodes and feeding of spores to infected animals, subsequently hatching within the dung. This effect is localized to early larval stages, as nematode egg output remained unaffected in infected animals fed fungal spores, while culture of infected feces yielded lower numbers of nematode larvae (Ahmed et al., 2014). Endoparasitic fungi, particularly *Harprosporium leptospira*, alongside nematode‐trapping fungi, can rapidly naturally colonize dung pats following deposition, with 83% and 58% of dung pats containing nematophagous fungi after 3 days in February and April, respectively, in a plot trial in New Zealand (Hay et al., 1997).

Nematode‐trapping fungi are the best documented fungal group with regard to their interactions with animal parasitic nematode populations, with *Duddingtonia flagrans* the main model species and candidate for biocontrol (Buzatti et al., 2015; Chauhan et al., 2005; Healey et al., 2018; Mendoza‐de‐Gives et al., 2018; Terrill et al., 2004). The presence of this species results in population reductions in a diverse array of animal parasitic nematodes, infecting many different host species (Bilotto et al., 2018; Buzatti et al., 2015; Pérez et al., 2017; Terrill et al., 2004; Terry, 2013; Zegbi et al., 2021), although effects are inconsistent (e.g., Faessler et al., 2007). As a method of biocontrol, *D. flagrans* is typically administered orally as fungal spores (Aguilar et al., 2008; Braga et al., 2020; Fausto et al., 2021), with the spores passing into the feces and augmenting the density of fungi in dung. Other nematode‐trapping fungi, such as *Arthrobotrys oligaspora*, also reduce nematode populations (Wang, Meng, et al., 2014) and can be administered orally (Cai et al., 2017). Interestingly, nematophagous behaviors among fungi may be enhanced by larger ecological networks involving bacteria that can be found within dung, which facilitate a lifestyle switch for some species, such as *A. oligospora*, from a saprophytic to a nematode‐trapping form (Ulzurrun & Hsueh, 2018; Wang, Li, et al., 2014).

Interactions between different nematode‐trapping fungi have been investigated, with mixed effects reported. Waghorn et al. (2002) found the greatest reduction of parasitic nematode larval abundance in dung was achieved by combined fungal treatments, which is supported by other studies (Silveira et al., 2017; Vilela et al., 2012, 2013). Some combinations of fungi, however, have been reported to result in an antagonistic rather than synergistic effect in the reduction of animal parasitic nematodes. Combined usage of *C. rosea* and *D. flagrans* decreased predation against *Haemonchus contortus* larvae from 88.9% to 91.5%, respectively, when applied individually, and to 74.5% when combined (Silva, Braga, Mendoza‐de‐Gives, Uriostegui, et al., 2015), which may be a consequence of spore production antagonism between competing fungi (Silva, Braga, Mendoza‐de‐Gives, Millán‐Orozco, et al., 2015). *Arthrobotrys robusta* was found to produce higher nematode fecal egg counts in heifers when added to feed, compared to when *D. flagrans* or *Monacrosporium thaumasium* were used individually or together (Luns et al., 2018). Variable effects against different parasite species could explain some of the apparent inconsistencies between studies (Paraud et al., 2006). Some fungi are known to produce toxins that can be detrimental to nematode communities, including animal parasitic nematodes (Kwock et al., 1992; Soares et al., 2018; Zarrin et al., 2015), although this group has not yet been developed for use in biocontrol.

Apart from nematophagous effects, fungi can facilitate parasite transmission by aiding movement of infective stages from the dung onto pasture. The fruiting bodies of *Pilobolus* spp., for example, mechanically assist the dispersion of infective larvae of the trichostrongylid lungworm *Dictyocaulus viviparus* (McCarthy et al., 2022). This action has not yet been investigated, however, in relation to the presence of other naturally occurring or biocontrol fungi or anthelmintic use, nor have the ecological conditions favoring *Pilobolus* growth been determined.

Nematophagous fungi therefore clearly have negative effects on helminth free‐living stages in the dung compartment. Effects, however, occur against a background of diverse natural fungal communities, and it is recommended that this be taken into account in future studies. Complementary methods of action between fungi that utilize different mechanisms of nematode predation have only been recently explored (Vieira et al., 2019, 2020), where it was shown that combined use of *Arthrobotrys cladodes* (nematode‐trapping) and *Pochonia chlamydosporia* (ovicidal) resulted in reduced eggs per gram in fecal pats and greater bovine weight gain than the control or when either was used in isolation. It is also important to evaluate how fungal communities may be influenced by factors to improve nematophagous activity, such as the density of animal parasitic nematodes and other organisms in dung. Further research is needed on parasite species preferences and antagonistic or synergistic actions to fully elucidate how the nematophagous community functions within grassland ecosystems under natural and augmented conditions. Outcomes are also likely to vary with the presence, abundance, and species composition of other nematodes present in dung, including saprophytic, predatory, and plant‐parasitic species.

#### Earthworms and parasites

Mortality of parasite stages in dung might be increased as a result of ingestion by earthworms, but the more important process is removal of dung from the soil surface to deeper layers by some earthworm species. This makes it harder for motile and nonmotile parasites to find their way back to the surface to continue their life cycle. Positive effects on transmission potential are possible through dispersal by earthworms that void egesta on the soil surface and through protection of parasites from adverse environmental conditions through ingestion and translocation into soil. Observed outcomes vary widely, and applications of bioaugmentation with earthworms for parasite control at farm scale are lacking.

Earthworms are a diverse group of the soil macro fauna that serve important roles in recycling nutrients, improving hydraulic conductivity and facilitating the assimilation of organic matter by other soil organisms (Bhadauria & Saxena, 2010; Marinissen & Ruiter, 1993; Scheu, 1987; Wen et al., 2020). Considering their large biomass, they are dominant organisms within the soil compartment (Lavelle & Spain, 2002; Medina‐Sauza et al., 2019) and aggregate underneath dung pats (Bacher et al., 2018, 2020). These localized “hotspots” provide increased opportunity for interactions between earthworms and animal parasitic nematode communities that are present within the dung compartment.

While earthworms feed upon the dung in which parasitic nematodes are undergoing a portion of their life cycle (Bacher et al., 2018; Knight et al., 1992), the net effect is highly uncertain (d'Alexis et al., 2009; Grønvold, 1979, 1987; Molavi et al., 2020; Waghorn et al., 2002; Zazouli et al., 2021). Consumption of dung by earthworms has been suggested to result in direct mortality of nematodes during passage through the gut (d'Alexis et al., 2009; Demetrio et al., 2019; Grønvold, 1987; Ray, 2018; Svendsen et al., 2003; Waghorn et al., 2002), while the impacts of dung translocation are also likely to be negative by moving surviving nematodes deeper within the soil compartment. Given differences in diet and behavior, interactions between earthworms and parasitic helminths must be considered on a guild and species basis.

Earthworms can be divided into three major guilds based on their ecological niche occupation and behavior (Sims & Gerard, 1999). Epigeic species are soil‐surface earthworms that live outside of the mineral substrata, endogeic species are soil‐dwelling species that build horizontal burrows, and anecic species are vertical, deep burrowing species. Differences in guild or species abundance may help to explain differences in the observed effects of earthworm–parasite interactions. Thus, L3 larvae of the GINs, *H. contortus*, and *Trichostrongylus colubriformis* were reported to be reduced by 29% and 33%, respectively, by D'Alexis et al. (2009) and *Teladorsagia circumcincta* by 63% by Waghorn et al. (2002) by the augmentation of earthworm populations. The introduction of four anecic earthworms per plot, of the species *Aporrectodea longa*, in addition to providing a more diverse earthworm community may have increased dung consumption and, therefore, translocation of nematode communities (Schon et al., 2019), resulting in the reported higher GIN reduction for Waghorn et al. (2002) compared to d'Alexis et al. (2009), who used one representative epigeic and one representative endogeic species. Furthermore, some earthworm species are known to deposit ingested material as casts at the soil surface, which may help explain the only reported positive interaction between earthworms and GINs (Grønvold, 1979). Grønvold (1979) used two plastic tubs, approximately 224 cm^3^, to construct an earthworm treatment group containing 27 earthworms and a control group that contained no earthworms. A 500‐g dung pat was placed in the center of each container, and after 50 days there was a 15‐fold increase in GIN L3 recovered from the soil under the earthworm treatment group compared to the control. It was concluded from this experiment that the earthworms used within the experiment deposited most of their egesta at the soil surface, and this translocation might have increased larval availability at the soil surface. While the study does not detail environmental conditions, this positive effect could be related to earthworms protecting GINs from harsh external factors, as the study was conducted in the middle of the summer. Translocation of parasites into the soil compartment may therefore result in a positive or negative effect on transmission potential depending on environmental conditions. Many parasites can actively migrate between the soil and herbage compartments (Amaradasa & Manage, 2010; Rose & Small, 1985), although with a limited energy reserve in the case of GIN L3 larvae (Van Dijk & Morgan, 2011), and so successful larval migration to the herbage with sufficient energy to complete the GIN lifecycle may only be possible from limited soil depths. Consequently, the depth of dung burial may be important when considering interactions with different guilds and species of earthworm, as well as effects on the viability of GIN L3 that successfully return to the surface through active migration. Because GIN larvae can mature quickly under the right conditions and migrate actively from the dung pat, while dung burial by earthworms can take several weeks, their effect on dung could come after the critical period for the parasite life cycle; this will depend on the season and weather conditions.

Interactions between earthworms and parasites have also been investigated in relation to manure application and the vermicomposting process, with mixed reports. Earthworm, *Eisenia fetida*, presence was reported to reduce the abundance of unspeciated parasite eggs following the vermicomposting process (Molavi et al., 2020), while other studies found no significant effect (Zazouli et al., 2021).

On balance, the presence of earthworms appears to reduce the number of parasites reaching pasture as a result of dung burial and consumption, although effects vary. Specifically, the direction and size of effects of earthworms on helminths may be dependent on guild and species (Grønvold, 1979; Hoeffner et al., 2022; Waghorn et al., 2002). Consquences of agricultural practices and potentially bioaugmentation on earthworm populations and helminth transmission should take account of these differences, with anecic species most likely to produce a negative effect, especially when environmental conditions favor active migration of nematode larvae from dung. Future research to explore guild‐ and species‐level effects of earthworms upon GIN transmission is needed before solid recommendations can be given to managers or effect sizes incorporated accurately into transmission models.

#### Dung beetles and parasites

Beetle species that use animal feces as a food source for their offspring can move the dung into the soil, while the feeding activity of the larval stages alters the structure and composition of the dung and could assist or inhibit the development and survival of parasite stages found there. On balance, experimental evidence suggests that dung beetles tend to reduce the availability of parasites, specifically GIN larvae, on pasture, but effects are variable. Scaling this effect to farm level via bioaugmentation and using it to reduce reliance on other control tools such as anthelmintic treatment has not been tested.

The title of dung beetle is indicative of this organism's importance in the decomposition, assimilation, and interaction with dung and, by extension, the free‐living stages of the animal parasitic nematode community. Dung beetles are one of the most representative taxonomic groups of insects associated with agricultural farmland and exhibit a wide array of life history strategies that provide critical ecosystem services to soil and dung communities (Arellano et al., 2023; Hanski & Cambefort, 1991). Consequential to their ubiquity and function, dung beetles have high potential for the modification of parasite transmission through their activity in dung degradation and movement. Dung beetle diversity and abundance are often controlled by climatic conditions and ecological niche availability (Gómez et al., 2020); however, their common effects on various animal parasitic helminths through dung burial are well documented (Bryan, 1973; Chirico et al., 2003; Huerta et al., 2013; Martinez et al., 2018; Ryan et al., 2011).

Dung burial may negatively impact animal parasite populations by increasing necessary migratory distances to return to the soil surface and onto grass, with the effect size dependent on the depth of brood burial (Bertone et al., 2006; Gregory et al., 2015). The presence of dung beetles often results in a significant reduction in infective parasite larval populations on the surrounding herbage (Bryan, 1973; Chirico et al., 2003; Forgie et al., 2018; Sands & Wall, 2017; Waterhouse, 1974), although there appears to be a temporal component to this impact. Some literature has suggested or demonstrated a “time‐bomb” effect whereby an initial decrease of infective larval abundance on surrounding herbage is noticed due to the burial of dung and larvae. This is then followed by an eventual increase due to larval migration to the soil surface, which could be beneficial to larval communities that may otherwise be exposed to adverse conditions (Chirico et al., 2003; Coldham, 2011; Sands & Wall, 2017), although this effect is not always supported in the literature (Forgie et al., 2018). In contrast to the “time‐bomb” effect, it has been hypothesized that aeration of the dung by beetles may counteract potential unfavorable anaerobic conditions within the dung pat, aiding parasite development (Sands & Wall, 2017). This idea has been developed to suggest that the positive effects of dung beetles on infective larval abundance may be exclusive to a temperate climate, as aeration of dung pats in tropical climates could exacerbate desiccation (Mfitilodze & Hutchinson, 1988). These contrary effects are nonexclusive, with the net outcome on parasite transmission depending on the environmental conditions. While the exact mechanisms of interaction between dung beetles and nematode communities have yet to be fully elucidated, it does appear that translocation effects outweigh dung aeration, at least in temperate conditions, such that dung beetle presence generally causes a decrease in the total number of animal parasitic nematodes reaching the herbage.

Consumption of dung may also lead to the destruction of parasite larvae directly; however, dung beetle food preference and maximal particle size of ingestion vary from species to species (Grønvold et al., 1992; Holter et al., 2002). The size of ingested material that has been suggested as possible for dung beetles in the current literature ranges between 5 and 130 μm, with the majority of temperate grassland dung beetle species consuming the smaller particle sizes (Holter et al., 2002; Holter & Scholtz, 2007). Given that the size of strongyle eggs are typically around 90 μm in length and 40 μm in width (Bucki et al., 2023; Cuomo et al., 2012; Harvey et al., 2019), it is unlikely that consumption by temperate grassland dung beetle species has a large impact on parasite transmission.

Based on current evidence, it is reasonable to suggest that enhanced dung beetle activity will tend to decrease the availability of parasites, specifically GIN infective larvae, on temperate grassland. Caveats apply, however, including variable effects and weather dependency, and these should factor into consideration of programs to use dung beetles for sustainable agricultural solutions. Under certain conditions, dung beetles could enhance transmission, such as burial when enviornmental conditions are detrimental to development and survival. Given that dung beetle burial is typically between 10 and 20 cm for common temperate grassland species (Snell‐Rood et al., 2016), this would be an achievable distance for gastrointestinal nematodes to migrate back to the soil surface (Fincher & Stewart, 1979). Sequestration of parasite stages within dung for later appearance on pasture might itself be beneficial to their control, for example, buying time for parasite evasion by livestock through rotational grazing or acting as a source of refugia for drug‐susceptible genotypes (Hodgkinson et al., 2019). Studies on effects at the farm and landscape levels are needed to properly assess the potential for dung beetle augmentation as a nature‐based or biocontrol intervention, as well as the consequences of nontarget effects of anthelmintics on them (de Souza & Guimarães, 2022; Jacobs & Scholtz, 2015; Manning et al., 2018). Furthermore, farm‐level studies that explore the abundance, succession, and longevity of dung beetle augmentation may also be important, to consider the feasbility of increasing dung beetle populations. This would help to consider the implications of resource competition, the maintenance of an effective dung beetle population for parasite control, and the implications across a farming season that experiences a multitude of different climatic conditions. On current evidence, it is reasonable to suggest that encouraging a healthy dung beetle population is likely to ameliorate rather than worsen parasite management on temperate pastures, but there is no solid basis to predict the size or consistency of this effect or its ability to replace other management tools such as anthelmintics.

### Nonparasite biotic interactions

While the focus of this review is interactions between parasites and biotic factors, interactions between free‐living taxa could indirectly affect parasites by modifying the abundance and activity of organisms directly impacting the life cycle, while bioaugmentation could have nontarget effects on soil communities. A thorough review of such second‐order interactions is beyond the scope of this paper but some examples are given below.

#### Fungi and earthworms

Fungus–earthworm interactions cover an array of direct and indirect mechanisms including arbuscular mycorrhizal fungi (AMF), so fungus–plant interactions (Paudel et al., 2016; Wang et al., 2021; Zaller et al., 2011), and the breakdown of organic matter and consequences for soil fertility (Aira et al., 2008; Gómez‐Brandón et al., 2011; Haitoa et al., 2018; Wu et al., 2017). Earthworm–fungus interactions are generally positive (Gómez‐Brandón et al., 2012), with the composting activity of epigeic earthworms increasing fungal mass (Aira et al., 2006, 2008; Chauhan, 2014; Gómez‐Brandón et al., 2011; Haitoa et al., 2018; Lazcano et al., 2008; Sharma et al., 2017; Srivastava et al., 2011; Wu et al., 2017) and earthworms grazing on fungi (Cooke, 1983; Moody et al., 1995), potentially increasing growth (Kaushik et al., 2012). While digestion of ingested fungal spores has been noted (Schönholzer et al., 1999), egested material has also been reported to leave fungi unaffected (Gómez‐Brandón et al., 2011; Pedersen & Hendriksen, 1993), and earthworms could therefore assist fungal dispersal. Differences in the transit of different fungal species through the earthworm gut have been reported (Curry & Schmidt, 2007; Tiunov & Scheu, 2000a, 2000b), but the effects of earthworms on the persistence and dispersal of fungal species used in the biocontrol of livestock helminths are not reported. Earthworm casting was greater in plots with feces treated with *D. flagrans*, however (Yeates et al., 2007), suggesting that bioaugmentation could affect earthworm activity, with unknown future effects. Application of *D. flagrans* does not appear to negatively affect earthworms through internal or external mycosis (Grønvold et al., 2000).

#### Fungi and dung beetles

Few studies report interactions between fungi and dung beetles (Figure 2). Dung beetles have been shown to both increase and decrease fungal growth in residual dung pats and brood balls (Lussenhop et al., 1980; Ykoyama et al., 1991), perhaps due to increased aeration coupled with desiccation. Dung beetles have been reported to feed on fungi (Halffter & Halffter, 2009; Holter et al., 2009) and to affect fungal communities (Lussenhop et al., 1980; Ykoyama et al., 1991). Regarding the nontarget effects of nematophagous fungi, *D. flagrans* did not impact the development of the dung beetle species *Aphodius constans* even at high concentrations (Paraud et al., 2007).

#### Earthworms and dung beetles

Earthworm–dung beetle interactions are characterized by a mixture of resource competition, whereby the presence of each group limits the net removal of dung by the other (O'Hea et al., 2010; Rosenlew & Roslin, 2008), and facilitation by succession, in which early colonization of dung by dung beetles attracts earthworms (Bacher et al., 2018; Gittings et al., 1994; Holter, 1979). Spring–summer peaks in dung beetle activity in temperate areas and decreased earthworm activity in dry summer conditions (Bayley et al., 2010; McDaniel et al., 2013; Storey & Storey, 2010; Tilikj & Novo, 2022) likely separate the relative importance of these groups by season. When modeling impacts of dung removal into soil on helminth transmission, it would seem pertinent to modify removal rates to account for this interaction especially in spring and autumn.

### System‐level interactions

Interactions between three or more of the biotic factors (taxonomic groups) considered here were rare, with only one paper detailing interactions in a primary study (Waghorn et al., 2002). That study aimed to explore how combinations of fungi, earthworms, and dung beetles impacted parasitic GIN L3 recovery from surrounding herbage, through a factorial experimental design at two different time points. When considering species in isolation, they found that earthworms reduced the total number of larvae recovered; dung burial, to simulate tunneling dung beetle activity, increased total larval recovery; and fungi had differential effects on total larvae recovery based on fungal species, fungal combinations, and the time point explored. When system‐level interactions were explored; however, they found that the negative effect of earthworms on total larval recovery was nullified when the dung was buried to a depth of 5 cm by hand. They also found that fungal presence reduced the positive effect that dung burial had on larval recovery (Waghorn et al., 2002).

Given the solitary study considering these complex interactions in the field, there is minimal research to consider how these interactions may occur within different locations or climates or with different species. Nevertheless, the results obtained by Waghorn et al. (2002) lead to a number of interesting questions that should be tested in future experiments to help elucidate the impacts of multispecies interactions on parasite free‐living stages. Interaction outcomes of free‐living parasite stages and faunal groups may be determined temporally. Faunal groups that interact with the dung first may have a larger influence on the outcome or direction of the effect on parasite free‐living stages compared to faunal groups that interact later in succession. It was found that earthworm activity was largely nullified upon dung burial (Waghorn et al., 2002), which could indicate that the succession of arrival and activity could influence the impact of other faunal groups. Therefore, faunal groups that exhibit an impact first may have a greater influence on GIN transmission than faunal groups that arrive later within succession. It is also important to consider that there may be a hierarchal ladder of effects, and constructing this formally could help determine the outcome of species interactions upon parasite transmission. The presence of some species may dominate the net effect of interactions with parasites, potentially linking the presence or absence of certain species with transmission outcomes. This could be particularly useful when considering parasite management strategies, as environmental surveys to understand what species are present could help to determine what nature‐based methods may be most suitable to combat parasite transmission. Despite the importance of multispecies interactions on parasite transmission, there is currently a void of literature that considers this effectively, which is surprising given the increase in support for future biocontrol and sustainable agricultural practices, which may rely on these fundamental ecological interactions for successful and effective application. Considering that the environmental stages in gastrointestinal parasites are subject to a wide array of grassland organisms, and their interactions with these faunal groups may be responsible for part of their development and survival, future research on these biotic interactions should be encouraged (Table 1).

**TABLE 1:** Summary of interactions between parasites and biotic factors.

Interaction	Positive mechanism	Negative mechanism	Key references	Stage of schematic influenced
Parasites and ovicidal fungi	None identified	Impaired development	Arroyo et al. (2017)	Egg
Parasites and hematophagous fungi	None identified	Nemtode‐trapping, hyphal capture	Waghorn et al. (2002), Fausto et al. (2021)	L3
Parasites and endoparasitic fungi	None identified	Fungi penetrate nematode cuticle	Ahmed et al. (2014), Rodríguez‐Martínez et al. (2018)	L1, L2, L3
Parasites and toxin‐producing fungi	None identified	Fungi produce nematotoxic enzymatic secretions	Zarrin et al. (2015), Soares et al. (2018)	L3
Parasites and earthworms	Translocation	Consumption, Translocation	Grønvold (1979), Waghorn et al. (2002), d'Alexis et al. (2009)	Egg, L1, L2, L3
Parasites and dung beetles	Improved aeration, favorable development conditions (time/temperature dependent)	Translocation, enhanced unfavorable conditions (time/temperature dependent)	Sands and Wall (2017), Forgie et al. (2018)	Egg, L1, L2, L3

*Note*: Collated information on the binary interactions between parasites and biotic factors that may influence transmission. Mechanism of actions are stated for either positive or negative interactions, with select key references provided. Interactions are considered against life cycle stage and can be read alongside the schematic, Figure 1, to visualize how each factor may influence parasite transmission.

### Influence of climate and abiotic factors on biotic interactions

Abiotic conditions are well known to influence the environmental stages of gastrointestinal parasites and could also influence the biotic interactions described above. Increased time within the dung pat as a result of slower development at cool temperatures, for example, may expose the stages to prolonged activity from biotic factors within the dung, which themselves may also have different responses according to prevailing climate and weather. The impact that abiotic factors, principally temperature and moisture, have on relevant faunal groups is therefore an additional factor in system behavior.

Increasing temperature tends to accelerate parasite development outside the host, while moisture is needed for development within the dung and free water from rainfall for migration out of dung onto herbage (Ciordia & Bizzell, 1963; Jehan & Gupta, 1974; Morgan & Van Dijk, 2012; O'Connor et al., 2007, 2008; Pandey et al., 1989; Wang et al., 2022).

Nematode‐trapping fungal activity has also been reported to increase with temperature, up to a threshold, with larval abundance taking over from temperature as the key limiting factor at higher temperatures (Buske et al., 2013; Fernández et al., 1999; Paraud et al., 2006). Increased stochastic variation in temperature (García‐Carreras & Reuman, 2013) has been shown to increase nematode‐trapping efficacy for *D. flagrans* compared to constant temperatures (Fernández et al., 1999) and alters nematode species preferences by fungi (Paraud et al., 2006), consistent across a changing climate. Humidity favors fungal survival, but nematode trapping increases in dry conditions (Faedo et al., 2002; Liu et al., 2009). High rainfall could also drive the breakup of dung and passive dispersal of parasite larvae from dung, reducing local larval abundance and, hence, the action of nematophagous fungi whose trapping activity is triggered by parasitic larval abundance (Buske et al., 2013). Earthworm feeding is reduced at temperatures below 10°C, and the development of immature earthworms and cocoon production halts completely above 40°C (Edwards & Bohlen, 1996). Reduced activity and abundance with increasing temperature is related to reduced soil moisture (Eisenhauer et al., 2014), which at extreme levels leads to aestivation or diapause (Bayley et al., 2010; Díaz Cosín et al., 2006; Holmstrup, 2001; Wever et al., 2001), while moderate increases in temperature may enhance earthworm activity, provided soil water content is sufficient (Eriksen‐Hamel & Whalen, 2005; Perreault & Whalen, 2006). Moisture is beneficial to earthworm communities, with higher burrowing activity often associated with wetter soils (Wen et al., 2020) and anecic earthworms themselves decreasing soil water content loss (Ma et al., 2020). For a discussion of how climate change might affect earthworms, see Singh et al. (2019).

Dung beetles appear to be less sensitive to temperature than fungi and earthworms, with large species able to self‐regulate temperature (Gómez et al., 2020; Mena, 2001; Verdú et al., 2006). Temperature has been shown to affect dung burial and decomposition in the presence of dung beetles, but reported effects varied widely (Gotcha et al., 2022; Holley & Andrew, 2018; Wu et al., 2011), perhaps due to species differences (Gotcha et al., 2020, 2021). Heavy rainfall accelerates the physical dispersal of dung and could reduce the relative importance of dung movement through beetle activity and, thus, their impacts on parasite larvae (Sands & Wall, 2017). Unlike earthworms, dung beetles do not thrive in wet soils (Osberg et al., 1994; Sowig, 1995), and moist conditions may therefore also reduce the effect of dung beetles on dung pats and parasite transmission (Waghorn et al., 2002).

The effect of dung burial on parasites is likely to depend on abiotic conditions. Thus, dry conditions at the soil surface act against larval migration from the dung and onto herbage, and dung desiccation would trap and eventually kill parasite stages (Stromberg, 1997): Burial at this stage would therefore enhance survival in the protected, moist brood ball (Sowig, 1995). When conditions are conducive to parasite survival and migration onto pasture, on the other hand, burial would present an additional barrier to successful movement onto herbage. Increased dung beetle activity may also increase the perforation of the pat and enhance tunnel formation, with aeration initially favoring parasite development but subsequently increasing the rate of desiccation (Penttilä et al., 2013). These contrary effects might explain the inconsistent results of field experiments and observations that seek to determine the effects of dung fauna on parasitic larval availability, as described above.

A changing climate may alter the importance of different interactions within the schematic framework through unequal effects upon individual taxa and processes. The effects of different climatic events and weather patterns are hard to predict but can be considered using the schematic and carried forward in extensions of parasite transmission models aiming to evaluate the effects of climate and climate change on infection patterns (Verschave et al., 2016; Wang et al., 2022), which explicitly consider the additional effects of biotic interactions and their potential to contribute to successful strategies for animal health on future farms.

#### Key gaps and future work

Harnessing the biological interactions described here has the potential to support parasite management. Biocontrol is becoming increasingly important for future effective, sustainable agricultural practices and would be useful as a tool to manage livestock parasites given the rise in anthelmintic‐resistant parasite populations, consumer demand for chemical‐free animal production lines, and societal pressure for nature‐based solutions. Changes in agricultural practices motivated by factors other than parasite control, including increased carbon sequestration, also have the potential to affect parasite transmission through these biotic interactions. This review details the many mechanisms and interactions to which the free‐living stages of helminth parasites, mainly gastrointestinal nematodes parasites of ruminants, are exposed. We also consider how abiotic factors, specifically climate, may influence these interactions. The aim is to highlight different avenues for integrated interventions in the hope of enhancing future nature‐based solutions for parasite management, as well as defining a framework for these interactions to assist the identification of important knowledge gaps and helping researchers to set focused studies in the wider ecological context.

A major gap in the literature concerns ecological interactions in the dung and soil, which could affect outcomes of biocontrol or bioaugmentation. Among the taxa reviewed, fungi are most advanced as a biocontrol option, with nematophagous fungal spores already available in some countries as a feed supplement, augmenting fungal populations in dung and, hence, their negative effects on parasites. Outcomes, however, have typically been evaluated while ignoring biotic interactions that are universal in real farm environments. Interactions between ovicidal and nematophagous fungi can have synergistic negative impacts on nematode larvae, increasing overall predation when compared to either fungus group individually (Vieira et al., 2019). On the other hand, fungi that utilize a similar mechanism to target identical life stages, such as different species of nematode‐trapping fungi, may compete with one another for resources and have negative impacts on the other (Ayupe et al., 2016). Dung beetle larval size has been shown to be negatively impacted by earthworm presence (Xie et al., 2020) due to the ingestion of beetle brood matter and resource competition. Conversely, parasitic nematode larval reduction by earthworm activity has been shown to be eliminated if dung is buried to simulate the activity of some dung beetle species (Waghorn et al., 2002). Earthworm and dung beetle interactions may therefore be ecotype‐ and species‐specific, varying based on burial depth, dung movement quantities, and abundance of each group, as well as the time course of dung burial by each group and how it relates to parasite development. Earthworms and dung beetles also play important roles in the dispersal of nematophagous fungi that may aid in the capture of parasitic L3 (Edwards, 2004). Environmental stages of GIN transmission are therefore subjected to interactions with complex ecological networks, which may vary spatiotemporally and geospatially along with the ecological communities. These have barely been investigated despite their obvious potential to affect the success, magnitude, and sustainability of interventions that seek to increase populations of beneficial parasite‐reducing taxa.

The influence of climate and weather on the impacts of fungi, beetles, and earthworms on parasites has been noted in individual studies but not explored systematically or carried forward to predict outcomes and provide advice on optimizing applications. Abiotic factors shape ecological communities and, upon changing, may also alter the ecological networks present (Kanianska et al., 2016; Slabbert et al., 2022), which may influence impacts on parasite stages. Abiotic factors may also play a role in modifying the effects of these organisms on parasite transmission, such as enhanced rainfall removing the impact that dung beetles have on parasitic L3 recovery (Sands & Wall, 2017). The importance of abiotic factors therefore may be crucially important to the outcomes of nature‐based solutions and biocontrol. Faster parasite development at higher temperatures gives less time for processes within the dung to impact populations, especially for this, like GINs that are able to migrate actively out of dung following development. Seasonality in activity could also influence outcomes on farms but is rarely considered in experimental work, which tends either to focus on peak periods of activity or provide nonseasonal artificial arenas: Alignment of dung removal with parasite development periods could greatly alter the magnitude of impacts on parasite epidemiology. Development of these lines of thinking is currently speculative given the lack of evidence. More experiments are needed that manipulate climatic factors directly in combination with bioaugmentation, as opposed to only describing variable outcomes under different weather conditions.

While repeating experiments across a wider range of conditions will be useful, much could be gained by focusing on process, for example, dung removal or larval killing and quantifying how that process affects parasites under different conditions. Bioaugmentation or mesocosm experiments could then determine effect sizes for the process at hand. Together, improved information on these processes and the parameters that shape them under different circumstances could be used to predict the effects of altered invertebrate diversity and abundance on parasite epidemiology under new and future conditions and guide hypotheses to reduce key uncertainties. Combining this information with knowledge of parasite population dynamics, specifically by incorporating the soil compartment and processes driven by biotic interactions (Figure 1) into existing modeling frameworks (e.g., Rose et al., 2015), seems an obvious way forward.

The range of helminths evaluated in past studies is broad, but with a strong bias toward GIN, whose free‐living stages are motile and more susceptible to some biotic influences, for example, nematode trapping, than to others, for example, by escaping from burial. Other helminths, present in feces and soil as immotile eggs, will respond differently. Since farmers must deal with a multitude of parasite species on livestock farms, differential effects on them could alter the parasitological landscape and management priorities. An attempt to systematically define the parasite life cycle and life history characteristics that influence impacts of different potential biocontrol agents (or the processes they engender) would help to predict the situations in which control is more or less likely to succeed and help optimize ultimate application.

Perhaps the biggest limitation of past work on the effects of biotic interactions is scale. Many experiments have been conducted at laboratory, mesocosm, and field plot scales, but almost none at whole‐farm or landscape scale. Larger studies are admittedly not easy to conduct, but without them it is impossible to translate effect sizes of reduced parasite availability, for example, to expected impacts on parasite transmission at herd level. This is perhaps particularly so for highly mobile taxa such as dung beetles, for which the benefits of bioaugmentation might also be difficult to sustain. Given that the application of biological solutions is likely to take place alongside and integrated with continued anthelmintic use on most farms, nontarget effects of anthelmintics such as on dung beetles should also be measured at the farm scale and strategies devised to avoid them.

In conclusion, biotic interactions affect parasite development and availability in temperate livestock pastures, and fungi, dung beetles, and earthworms show promise as tools for sustainable parasite management using biocontrol or bioaugmentation. The existing literature provides strong proof of principle at the experimental level but much less so at farm scales. Further research is needed to rectify this and to better understand the biotic and abiotic interactions that shape ecological communities and their effects on parasite transmission. A more rigorous definition of processes and the estimation of effect sizes could be usefully integrated with models of parasite population dynamics to scope and refine applications and define hypotheses and priorities for future work.

## CONFLICT OF INTEREST STATEMENT

The authors declare no conflicts of interest.
